# Comment on Bertoldo et al. Definition, Assessment, and Management of Vitamin D Inadequacy: Suggestions, Recommendations, and Warnings from the Italian Society for Osteoporosis, Mineral Metabolism and Bone Diseases (SIOMMMS). *Nutrients* 2022, *14*, 4148

**DOI:** 10.3390/nu15030498

**Published:** 2023-01-18

**Authors:** Sunil J. Wimalawansa

**Affiliations:** Endocrinology and Nutrition, Department of Medicine, Cardiometabolic and Endocrine Institute, North Brunswick, NJ 08873, USA; suniljw@hotmail.com

We are responding to a report by Bertoldo et al. in October 2022 in *Nutrients* [1]. The authors’ definitions used (Table 1) are outdated: only applicable to preventing rickets in children and osteomalacia in adults, not to the extraskeletal benefits of vitamin D. They failed to clarify this.

The authors’ recommendations apply only to calcium—bone: musculoskeletal field. Therefore, generalization outside of this is inappropriate and misleading. The authors stated:

1. Suggest dose of cholecalciferol supplementation 800–2000 IU/day.
2. Recommend ‘loading’ dose, cholecalciferol 3000–10,000 IU/day (average 5000 IU/day) for 1–2 months.
3. Suggest supplementation with cholecalciferol at least 2000 IU/day in patients with severe hepatic insufficiency.

(1) Suggested vitamin D doses are adequate to avoid rickets and osteomalacia but grossly insufficient for nonskeletal disorders: e.g., maintenance of a robust immune system requires approximately 5000 IU per day for non-obese 70 kg adults [2]. Those taking agents that increase the catabolism of vitamin D (e.g., anti-epileptic or ant-retroviral) or are obese require two to four times this daily dose [2].
(2) Recommended <10,000 IU (5000/day) is not a loading dose, but within usual daily doses. Most vitamin D scientists and physicians recommend upfront loading doses between 100,000–400,000 IU as single or divided doses over a few days to raise serum 25(OH)D rapidly and to fill the body storage in deficient persons [3].
(3) The suggested cholecalciferol (D_3_) dose of 2000 IU/day is ineffective and insufficient. Those with hepatic failure cannot 25-hydroxylate vitamin D in the liver; therefore, giving them cholecalciferol is futile. Instead, they should be given 25-hydroxylated vitamin D, calcifediol [25(OH)D] to bypass the liver: administered, based on their body weight (0.014 mg/kg), approximately once a week.

The authors’ conclusion stated, “this position statement is for use by clinicians to face the issues of the definition, assessment, and management of vitamin D inadequacy, in order to (a) improve and standardize the “clinical practice,” (b) offer the patient for “best care”, to be followed uniformly at national level, and (c) guarantee an evidence-based reference for regulatory organizations and payers.”

However, (a) the flawed definitions of vitamin D deficiency included do not help clinicians or patients and are not accepted by most scientists and clinicians, (b) said recommendations are misleading and do not improve the standard of care, and (c) recommendations are not evidence-based, partly due to the limited and biased references used: thus they are inaccurate. 

The authors concluded that data did not support vitamin D treatment outside the hospital to prevent fractures and recommended not prescribing vitamin D supplements to the general population. This statement and explanation are incorrect, regressive, and misleading.

Among thousands of publications the authors failed even to acknowledge over 200+ published clinical studies, including over 40 randomized control clinical studies on the beneficial effects of vitamin D in overcoming COVID-19, over the past two years alone [2,4]. As with the definitions provided (Table 1), the recommendations and conclusions by Bertoldo et al. [1] are also outdated and misleading [2].

While there are negative studies [5], most randomized control clinical studies, meta-analyses, and other clinical studies using vitamin D with proper study designs using correct doses for an adequate duration in vitamin D deficient subjects, reported favorable clinical outcomes [6] for both musculoskeletal and extraskeletal systems, especially the immune system [7,8,9,10]. Generalizing limited musculoskeletal effects of vitamin D with broader conclusions and recommendations based on ‘narrow’ definitions focused on bone, is flawed. If clinicians use authors’ recommendations, such misguidance can harm patients.

Despite unwarranted broader conclusions, the review did not encompass the broader benefits of vitamin D in those with hypovitaminosis D [7]. The authors should have specified this in the abstract and conclusions: in the absence, the article adds confusion but little value. 

## Figures and Tables

**Table 1:** ‘*Definitions*’ of Vitamin D Status used (data are grossly outdated).

	Deficiency	Insufficiency	Optimum
General population	<10 ng/mL	<20 ng/mL	20–50 ng/mL
Population at risk/Treatment	<10 ng/mL	<30 ng/mL	30–50 ng/mL
